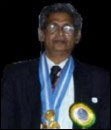# President's Message

**DOI:** 10.4103/0019-5049.60488

**Published:** 2010

**Authors:** J Ranganathan

**Affiliations:** President, ISA, National Email: drjrsalem@gmail.com

Honourable members of ISA,

At the outset, I thank all the members of the fraternity of Anaesthesiology, who helped raise this noble profession of “Alleviating Human Sufferings” to this stature. The interventional procedures, which were thought impossible in last century, could be accomplished due to the dedication, devotion and academic-cum-scientific inputs by anaesthesiologists across the world under the banner of World Federation of Societies of Anaesthesiologists (WFSA). Indian Society of Anaesthesiologists (ISA) has the distinction to be the founder member of WFSA and has immensely contributed to the development of this faculty. I, as President, ISA, humbly shoulder the responsibility to further elevate it to a greater height.

India is the largest democracy of this world with heterogeneous economic, social, linguistic and religious culture. To satisfy the professional needs of the citizens, ISA has prepared a long agenda. For this, Vision 2020 has been enumerated and steps have been taken to achieve the same. However, some points need urgent attention for which the Honourable members of the Governing Council of ISA, with their collective wisdom, have put forward the following few points before you and the authority (health service providers, both in Government and NGOs including PPP).

There is a common belief in the Government sector that there is a shortage of anaesthesiologists in emergency obstetric care in a rural setup. This is a misconception. There is ill distribution of all the medical specialists between urban and rural areas. Anaesthesiology is not the sole faculty. Emergency obstetric care can be provided by utilizing nearby anaesthesiologists. Efficient antenatal care can categorize high risk cases. Necessary prior action plan, including referring high risk cases to higher health-care centres, can be worked out. Remuneration to on-call anaesthesiologists must be immediate, without delay on the pretext of fulfilling government paper formalities (which causes failure of most government programmes).

These anaesthesiologists can be utilized for other cases including trauma and emergency care. Proper protocol has to be formulated to provide quality health care by public-private-participation (PPP). Regular evaluation and timely modification of these protocols according to local need will ensure greater compliance.

There must be a mandatory provision for post-graduates in all specialties to have minimum period of rural service before final specialist registration by IMC (in the line of compulsory rotating internship).

There must be definite teaching curriculum for DA, DNB and MD. This will ensure trained working professionals as well as academicians for further research and expertise in super-specialties.

The anaesthesiologists are always under stress. Hence they are susceptible to different professional health hazards, which are well documented. Hence ISA has mooted a self-help scheme in the form of Family Benevolent Fund (FBF). All the members of ISA are requested to enrol in this unique scheme, the details of which can be availed from ISA website or ISA National office.

In the electronic era of 21^st^ century, ISA has already worked out the modalities for electronic/on-line voting. Members will have the opportunity to upload their own details and avail all the necessary details through information-highway.

To conclude, I am to state that the memorandum is long. I, as President ISA and my fellow Governing Council members, along with due advice and feedback from our learned members will keep no stone unturned to deliver efficient cost-benefit service to the wide-spectrum of population of India.

I extend a warm greeting to all my colleagues and wish all the success in their socio-academic voyage.

Thanking you,

LONG LIVE ISA: JAI HIND